# Upper gastrointestinal endoscopy in the patient population of Kumasi, Ghana: Indications and findings

**DOI:** 10.11604/pamj.2014.18.327.4806

**Published:** 2014-08-25

**Authors:** Adam Gyedu, Joseph Yorke

**Affiliations:** 1 School of Medical Sciences, Kwame Nkrumah University of Science and Technology, Kumasi, Ghana; 2 Directorate of Surgery, Komfo Anokye Teaching Hospital, Kumasi, Ghana

**Keywords:** Upper gastrointestinal, endoscopy, Kumasi

## Abstract

**Introduction:**

Characteristics of patients undergoing Upper GI endoscopy (UGIE) in Kumasi, Ghana are largely unknown. This paper reviews the work of three endoscopy units in Kumasi.

**Methods:**

A review of the records of patients undergoing diagnostic UGIE in the three centers from October 2006 to December 2011 was undertaken.

**Results:**

3110 completed UGIE were performed over the period. In 80% of the patients the primary indication for UGIE was dyspepsia occurring without any other symptom. In 50% of patients UGIE findings were normal. Peptic ulcer disease, the most common positive finding, was diagnosed in 27.4% of patients. The odds ratio (O.R) of yielding a positive endoscopic finding for patients presenting with recurrent vomiting, dyspepsia associated with weight loss and UGI bleeding were 3.87 (95% C.I: 2.23-6.69), 1.72 (95% C.I: 1.03-2.87) and 1.81 (95% C.I: 1.03-3.16) respectively. Dyspepsia without any other symptom, on the other hand, yielded a positive endoscopic finding with O.R of 0.67 (95% CI: 0.57-0.80). Alarm symptoms (UGI bleeding, recurrent vomiting, dysphagia or weight loss associated with dyspepsia) yielded a positive endoscopic finding with an O.R of 2.34 (95% C.I: 1.74-3.13).

**Conclusion:**

Most patients in Kumasi underwent UGIE because of dyspepsia in the absence of any other symptom. These patients were more likely to have normal endoscopic findings. The opposite was true for those presenting with alarm symptoms. Our results suggest that initial UGIE may be preferentially offered to patients presenting with alarm symptoms especially in resource-poor settings such as ours.

## Introduction

Upper Gastrointestinal (UGI) symptoms are among the commonest complaints for which patients seek medical attention, with the annual prevalence of dyspepsia approximating 25% [1]. Diseases associated with these symptoms are leading causes of morbidity and mortality globally. Peptic ulcer disease (PUD), gastroesophageal reflux disease (GERD) and cancers affect millions of people worldwide [2]. Endoscopy holds an important place in the diagnosis of UGI conditions by enabling visualization, photography, ultrasonography, and biopsying of suspicious lesions [3]. It gives a better diagnostic yield over radiology particularly in the investigation of upper gastrointestinal bleeding, inflammatory conditions of the upper gastro-intestinal track like esophagitis, gastritis and duodenitis as well as the diagnosis of Mallory Weiss tears and vascular malformations [4].

In many cases, accurate diagnosis of pathology can lead to timely, focused treatment. In Ghana, upper gastrointestinal endoscopy (UGIE) service is offered in the three teaching hospitals and a few other public or private centers. Previously published work involving UGIE among the Ghanaian population have only included patients from Accra [4–6]. Characteristics of patients undergoing UGIE in Kumasi are largely unknown. The aim of this study was to document the common UGIE findings among the patient population of Kumasi.

## Methods

This is a retrospective review of the records of all patients who underwent diagnostic UGIE in three endoscopic centers in Kumasi from October 2006 to December 2011. This is a period when there was a break in service of the diagnostic center at Komfo Anokye Teaching Hospital (KATH), the main referral hospital for most of the northern half of Ghana, due to malfunctioning equipment. The centers are Soyuz, Bestcare and Histolab. All three centers operated on an open access policy. As such primary care providers could directly refer patients without consultation with a gastroenterologist. They served patients referred from various hospitals in Kumasi and its environs.

All patients underwent standard pre-procedure preparation that included an overnight fast while nasogastric drainage of the stomach was preferred for those with suspected gastric outlet obstruction. The main form of analgesia and sedation used were throat spray with 2% lignocaine (Xylocaine^®^, Astra-Zeneca, UK) and intravenous sedation with midazolam (Dormicum^®^, Roche, Switzerland) respectively. All reports were included except those that were grossly incomplete or inconsistent. Data extracted from the records included the age and gender of the patients, principal indication for the procedure and primary UGIE findings. Although biopsies were routinely taken from lesions in the stomach and esophagus for histology, information on histological diagnosis was not available.

Data entry and analysis was done with Stata version 11 (StataCorp, College Station, TX). Descriptive statistics were expressed as frequencies and percentages. Alarm symptom was defined as UGI bleeding, recurrent vomiting, weight loss associated with dyspepsia or dysphagia. The likelihood of yielding a positive finding on UGIE for various indications was expressed as odds ratios (O.R) with 95% confidence intervals (C.I). The association between alarm symptoms and various UGIE findings was also expressed as O.R with 95% C.I.

## Results

Table 1 summarizes the characteristics of the study population. Three thousand one hundred and ten patients underwent complete UGIE over the period. 57.3% of the patients were female. Most patients (36.2%) were in the 20-39 age group. 2.1% were over 79 years old. By far the most common primary indication for UGIE was dyspepsia occurring without any other symptom, accounting for almost 80% of the cases. For 3.5% of the patients the primary indication was GERD symptoms (retrosternal pain, regurgitation, heartburn or chest pain). Other notable primary indications were recurrent vomiting, dyspepsia associated with weight loss and UGI bleeding. In 11.1% of patients no indication was stated.

**Table 1:** Characteristics of study population (N = 3110)

Variable	Number of patients (%)
Age (year)	
<20	104 (3.3)
20-39	1127 (36.2)
40-59	959 (30.9)
60-79	394 (12.7)
>79	66 (2.1)
Unknown	460 (14.8)
**Sex**	
Female	1783 (57.3)
**Primary indication for UGIE**	
Dyspeptic symptoms alone	2435 (78.3)
GERD symptoms	107 (3.5)
Recurrent vomiting	76 (2.4)
Dyspepsia & weight loss	62 (2.0)
Upper GI bleeding	53 (1.7)
Dysphagia	32 (1.0)
Not stated	345 (11.1)

Endoscopic findings are presented in Table 2. Half of the patients had normal UGIE findings and this was the most common finding. Peptic ulcer disease (PUD) defined as gastritis, duodenitis or both, was the most common positive finding. This was seen in 27.4% of all cases. Gastric ulcer (4.7%) was seen more frequently than duodenal ulcer (3.0%). Gastric cancer was found in 2.1% of cases and non-candidial esophagitis was found in 5.6%. Less commonly reported findings were esophageal varices, gastric polyp and distal esophageal cancer. Recurrent vomiting, dyspepsia associated with weight loss and UGI bleeding all had significantly increased odds of yielding a positive endoscopic finding. Dyspepsia without any other symptom, on the other hand, had significantly decreased odds of yielding a positive endoscopic finding (Table 3].

**Table 2:** Primary upper GI endoscopic findings (N = 3110)

Endoscopic findings	Number of patients (%)
Normal findings	1556 (50.0)
PUD	850 (27.4)
Gastric ulcer	147 (4.7)
Duodenal ulcer	94 (3.0)
Gastric cancer	66 (2.1)
Gastric polyp	23 (0.7)
Duodenal cancer	4 (0.2)
Duodenal diverticulum	2 (0.1)
Distal esophageal cancer	3 (0.1)
Esophageal candidiasis	50 (1.6)
Noncandidial esophagitis	174 (5.6)
Hiatus hernia only	91 (2.9)
Esophageal varices	37 (1.2)
Esophageal stricture	13 (0.4)

*PUD: gastritis, duodenitis or both

**Table 3:** Association between Indication for upper GI endoscopy and a positive endoscopic finding

Indication for endoscopy	Positive finding N = 1554*	Normal finding N = 1556*	O.R (95% CI)
Dyspeptic symptoms alone	1165 (75.0)	1270 (81.6)	0.67 (0.57-0.80)
GERD symptoms	50 (3.2)	57 (3.7)	NS
Sustained vomiting	60 (3.9)	16 (1.0)	3.87 (2.23-6.69)
Dyspepgia & weight loss	39 (2.5)	23 (1.5)	1.72 (1.03-2.87)
Upper GI bleeding	34 (2.2)	19 (1.2)	1.81 (1.03-3.16)
Dysphagia	19 (1.2)	13 (0.8)	NS

* Values are N (column%); NS: non-significant

The O.R's of diagnosing various conditions endoscopically in patients presenting with alarm symptoms compared to those presenting without alarm symptoms are shown in Table 4. While for PUD the O.R was 1.02 (95% CI: 0.75, 1.38), it was 8.17 (95% CI: 4.81, 13.85) for gastric cancer. Compared to those presenting with non-alarm symptoms the O.R of encountering normal findings on endoscopy in patients presenting with alarm symptoms was 0.43 (95% CI: 0.32-0.57). Gastric cancer was reported as the primary endoscopic finding in 66 patients. Patient age at diagnosis rose steadily to a peak at 40-59 years and then fell gradually (Table 5). Upper GI bleeding was the indication for endoscopy in 53 patients. PUD was the primary finding in 16 of them while in 19 patients the findings were normal. Esophageal varices, gastric ulcer and duodenal ulcer were found in five, five and two patients respectively.

**Table 4:** Upper GI endoscopic findings in patients with and without alarm symptoms

Endoscopic finding	Alarm symptom N (223)*	Non-alarm symptom N (2542)*	O.R (95% CI)
Normal findings	71 (31.8)	1327 (52.2)	0.43 (0.32-0.57)
PUD	61 (27.4)	686 (27.0)	NS
Gastric ulcer	16 (5.1)	116 (4.6)	NS
Duodenal ulcer	11 (4.9)	75 (3.0)	NS
Gastric cancer	24 (10.8)	37 (1.5)	8.17 (4.81-13.85)
Gastric polyp	4 (1.8)	16 (0.6)	2.89 (1.00-8.29)
Duodenal cancer	2 (0.9)	2 (0.1)	11.50 (2.02-65.38)
Duodenal diverticulum	1 (0.5)	1 (0.0)	-
Distal esophageal cancer	1 (0.5)	1 (0.0)	-
Esophageal candidiasis	4 (1.8)	42 (1.7)	NS
Noncandidial esophagitis	11 (4.9)	139 (5.4)	NS
Hiatus hernia only	1 (0.5)	71 (2.8)	0.16 (0-0.90)
Esophageal varices	9 (4.0)	23 (0.9)	4.61 (2.14-9.9)
Esophageal stricture	7 (3.1)	6(0.2)	13.70 (4.78-39.22)
Any organic disease	152 (68.2)	1215 (47.8)	2.34 (1.74-3.13)

* Values are N (column%); NS: non-significant; Alarm symptom: upper GI bleeding, recurrent vomiting, weight loss associated with dyspepsia or dysphagia

**Table 5:** Characteristics of patients with a primary endoscopic finding of gastric cancer

Age	Number of patients	Number of patients with gastric cancer (%)
<20	104	0 (0.0)
20-39	1127	12 (18.2)
40-59	959	23 (34.9)
60-79	394	19 (28.8)
>79	66	8 (12.1)
Unknown age	460	4 (6.0)
Total	3110	66 (100.0)

## Discussion

This study represents the first ever report on UGIE findings from Kumasi, Ghana. Previously published work involving UGIE among Ghanaian patients have all come from Accra [4–6]. Dyspepsia in the absence of any other symptom was the indication for UGIE in the vast majority of our patients. Such a high proportion has also been reported by the studies from Accra [5, 6] and a similar trend can be observed from other West African and East African studies [7–10]. Other common reasons for UGIE among our patients were GERD symptoms, recurrent vomiting and dyspepsia associated with weight loss. Only 1% of our patients underwent UGIE for dysphagia. Although dysphagia is less encountered in studies from West Africa, it accounts for 25-47% of indications for UGIE among East African studies [9, 11, 12].

Similar to previous Ghanaian studies [4, 6] PUD was the commonest endoscopic finding among our patients. Gastric ulcer was diagnosed more frequently than duodenal ulcers among our patient population in the ratio of 1.6:1. This is in contrast to the findings of one study from Accra that reported more duodenal ulcers than gastric ulcers in the ratio of 6.1:1 [4]. Ohene-Yeboah et al. have previously reported that gastric perforations are more common than duodenal perforations among the Kumasi population. They also noted that patients presenting with gastric perforations were more frequent users and abusers of non-steroidal anti inflammatory drugs and herbal medicines or concoctions [13]. Half of our patients had a normal endoscopic finding. A similar trend can be found in most studies published across Africa [4–6, 9, 12, 14, 15].

The primary aim of endoscopy in the management of patients with UGI symptoms is to detect organic disease. UGIE services, however, is not widely available in Ghana, and waiting times for the procedure are long [5]. Close to 80% of our patients presented with dyspepsia not associated with any other symptom and in 50% of the patients the endoscopic findings were normal. There is the need to be able to decide which patient should preferentially undergo the procedure, especially in our resource-poor setting.

From our data, patients presenting with dyspepsia in the absence of any other symptom were significantly less likely to get a positive endoscopic diagnosis. Those presenting with recurrent vomiting, dyspepsia together with weight loss or UGI bleeding were significantly more likely to get a positive diagnosis. Also, a patient presenting with an alarm symptom was 2.3 times more likely to yield a positive endoscopic finding compared to one without an alarm symptom. The odds ratio of the same patient having a normal endoscopic finding was actually less than one-half.

Gastric cancer was diagnosed, by endoscopy, about eight times more frequently in patients presenting with an alarm symptom. Patients older than 40 years made up about 75% of this group. From our data it seems prudent to initiate empirical treatment for patients presenting with dyspepsia in the absence of any other symptom and reserving UGIE for those presenting with any of the alarm symptoms, especially if they are older. This approach is supported by the recommendations of the American Gastroenterological Association [16]. H. pylori testing plays an important role in the current recommendations of the American Gastroenterological Association but was not routinely available at our study centers. As testing becomes increasingly available in Kumasi, it remains to be seen how emerging data will shape the management of dyspepsia in our setting.

A limitation of our study is the lack of confirmed pathology for patients with endoscopic diagnosis of cancer. This was because patients reported back to their referring doctors and not to the endoscopist. This could affect the number of cases categorized as either ulcers or malignancies. Also, because the study used data from private endoscopic centers a selection bias could be contemplated. Although these limitations exist, the paucity of data from Kumasi in the literature makes this report a useful contribution. Further work on UGIE findings among the Kumasi population must incorporate H. pylori testing and histologic diagnoses of biopsies.

## Conclusion

The most common indication for undergoing UGIE in Kumasi was dyspepsia in the absence of any other symptom. These patients were more likely to have normal endoscopic findings. Those presenting with alarm symptoms were more likely to have a positive endoscopic finding. Our results suggest that initial UGIE may be preferentially offered to patients presenting with alarm symptoms especially in resource-poor setting such as ours.
